# The Irish

**Published:** 1863-03

**Authors:** 


### THE IRISH.

Observant persons who have traveled abroad, will bear witness with us to the truthfulness of the old remark, that "An Irish gentleman is the most finished gentleman in the world." There is a vivacity, a frankness, a cordiality, and a high sense of honor about him, which will win a way into the heart.

Another item just as true is, that it is almost impossible to find in the United States an Irishman, who is strictly temperate as to liquor, who is not a man of substance and of influence. Nothing that we could say will so fully give the idea of the loss our own country sustains, and which the Irish race sustains, in consequence of their great besetting and besotting sin. So general is it, that it may be considered a national sin—the love of drink.

What young Irish hearts can do, before this love of drink takes possession, is indexed in the tens of thousands and scores of thousands of dollars which are sent, in a monthly stream, to the Old Country, by Irish girls, the savings of menial toil, to bring decrepid parents and helpless brothers and sisters to this happier land. What shall be done to save a nation capable of deeds like these, from the Demon of Drink? It is their greatest barrier to things and deeds greater than these.

